# P-39 - MEASURING PERFORMANCE OF SEARCH STRATEGIES FOR EVIDENCE SYNTHESIS IN PANDEMIC AND EMERGENCY PREPAREDNESS

**DOI:** 10.1093/pubmed/fdag046.084

**Published:** 2026-07-09

**Authors:** Dr Jennifer Hill, Ms Jodie Walker

**Affiliations:** UK Health Security Agency; UK Health Security Agency

## Abstract

**Background:**

To support incident response the UK Health Security Agency All Hazards Public Health Response evidence review team developed sleeper protocols on common review questions for emerging infectious diseases, including pre-prepared search strategies. Performance of these searches will be validated, starting with a search on disease incubation and infectious period.

**Objectives:**

1. To validate the pre-prepared search strategy on disease incubation and infectious period in Ovid Medline and Embase by determining sensitivity and precision.

**Methods:**

Existing systematic reviews on incubation and/or infectious period for any infectious disease will be identified via searches of Ovid Medline and Embase. Included studies will be extracted and screened by 2 independent human screeners, to create a validated reference set. Each study in the reference set will be searched in Medline and Embase to confirm whether it's available in those databases. Unavailable studies will be removed from the reference set. The incubation and infectious period search will be combined with the reference set in both databases to determine sensitivity (how many studies out of the total reference set are retrieved) and precision (the proportion of retrieved articles that are relevant).

**Results:**

The search will be validated at strategy and term level. 80% will be used as the threshold for acceptable sensitivity. If the search retrieves less than 80% of the reference set, search terms will be amended to increase sensitivity. Individual search terms will be checked to determine whether they increase results without retrieving any reference set items, therefore lowering precision unnecessarily. Details of diseases covered by reference set studies will be reported.

**Conclusions:**

Validation of the search block on incubation and infectious period will establish methods which can be applied to validate other sleeper search strategies, and will improve rigor of evidence syntheses which use this search by ensuring more comprehensive coverage of evidence.

